# Characterizing sensitivity and coverage of clinical WGS as a diagnostic test for genetic disorders

**DOI:** 10.1186/s12920-021-00948-5

**Published:** 2021-04-13

**Authors:** Yan Sun, Fengxia Liu, Chunna Fan, Yaoshen Wang, Lijie Song, Zhonghai Fang, Rui Han, Zhonghua Wang, Xiaodan Wang, Ziying Yang, Zhenpeng Xu, Jiguang Peng, Chaonan Shi, Hongyun Zhang, Wei Dong, Hui Huang, Yun Li, Yanqun Le, Jun Sun, Zhiyu Peng

**Affiliations:** 1 BGI Genomics, BGI-Shenzhen, Shenzhen, 518083 China; 2 Tianjin Medical Laboratory, BGI-Tianjin, BGI-Shenzhen, Tianjin, 300308 China; 3 Binhai Genomics Institute, BGI-Tianjin, BGI-Shenzhen, Tianjin, 300308 China; 4 BGI-Beijing Clinical Laboratories, BGI-Shenzhen, Beijing, 101300 China

**Keywords:** WGS, Sensitivity and PPV, DP and breadth of coverage, CNV, Clinical diagnosis

## Abstract

**Background:**

Due to its reduced cost and incomparable advantages, WGS is likely to lead to changes in clinical diagnosis of rare and undiagnosed diseases. However, the sensitivity and breadth of coverage of clinical WGS as a diagnostic test for genetic disorders has not been fully evaluated.

**Methods:**

Here, the performance of WGS in NA12878, the YH cell line, and the Chinese trios were measured by assessing their sensitivity, PPV, depth and breadth of coverage using MGISEQ-2000. We also compared the performance of WES and WGS using NA12878. The sensitivity and PPV were tested using the family-based trio design for the Chinese trios. We further developed a systematic WGS pipeline for the analysis of 8 clinical cases.

**Results:**

In general, the sensitivity and PPV for SNV/indel detection increased with mean depth and reached a plateau at an ~ 40X mean depth using down-sampling samples of NA12878. With a mean depth of 40X, the sensitivity of homozygous and heterozygous SNPs of NA12878 was > 99.25% and > 99.50%, respectively, and the PPV was 99.97% and 98.96%. Homozygous and heterozygous indels showed lower sensitivity and PPV. The sensitivity and PPV were still not 100% even with a mean depth of ~ 150X. We also observed a substantial variation in the sensitivity of CNV detection across different tools, especially in CNVs with a size less than 1 kb. In general, the breadth of coverage for disease-associated genes and CNVs increased with mean depth. The sensitivity and coverage of WGS (~ 40X) was better than WES (~ 120X). Among the Chinese trios with an ~ 40X mean depth, the sensitivity among offspring was > 99.48% and > 96.36% for SNP and indel detection, and the PPVs were 99.86% and 97.93%. All 12 previously validated variants in the 8 clinical cases were successfully detected using our WGS pipeline.

**Conclusions:**

The current standard of a mean depth of 40X may be sufficient for SNV/indel detection and identification of most CNVs. It would be advisable for clinical scientists to determine the range of sensitivity and PPV for different classes of variants for a particular WGS pipeline, which would be useful when interpreting and delivering clinical reports.

**Supplementary Information:**

The online version contains supplementary material available at 10.1186/s12920-021-00948-5.

## Background

Recently, whole-exome sequencing (WES) and whole genome sequencing (WGS) have been routinely used and are gradually being optimized for the detection of rare and common genetic variants in humans [1–4]. Several studies have compared the performance of WES and WGS in both technical aspects and clinical aspects [5, 6]. For example, focusing on mappability, Barbitoff et al. assessed the coverage of coding regions provided by several modern WES platforms and WGS [5]. Scala et al. compared the diagnostic performance of WES and WGS in children and adults with epilepsy [6]. In comparison to WES, WGS is more powerful for detecting variants. Theoretically, WGS has the potential to identify nearly all forms of genetic variation [7], including single-nucleotide variants (SNVs), in both the protein-coding and noncoding regions (such as introns and promoters) of the genome, small insertions/deletions (indels), and copy-number variants (CNVs) [8, 9]. Without target region selection, WGS could provide a more uniform DP for the genome, making the detection of CNVs easier. Moreover, WGS could provide higher sensitivity and a higher yield in variant detection in the coding regions [10–12]. Several studies have demonstrated the advantages of WGS for variant detection [13–16]. For patients with highly suspected genetic disorder, WGS might be optimal for further evaluation when the patient remains undiagnosed after clinical WES. With the reduction of the cost of sequencing and its incomparable advantages, WGS is likely to change the clinical diagnosis of rare and undiagnosed diseases and is bound to become a routine part of clinical care in the near future.

The analysis of a clinical WGS usually starts from quality evaluation. Mean DP is often recognized as a general indicator of overall sensitivity. It has been reported that variant calling is more reliable with increasing DP [17]. As a crucial factor in data quality, most centers conducting MPS technology would determine thresholds for average DP across WES/WGS and determine the minimum DP that must be achieved for a certain fraction of target bases [18]. The breadth of coverage describes the fraction of the total target genomic region that is sequenced to an adequate depth in a particular assay [4]. The American College of Medical Genetics and Genomics (ACMG) recommends that 90%-95% breadth of coverage above a minimum threshold of 10X should be achieved for exome data with an average depth of 100X [18]. Thus far, a mean depth of 30-50X is the most widely used mean DP for WGS [19, 20]. However, the sensitivity and breadth of coverage of clinical WGS have not been fully evaluated, especially for CNV detection, some of which are associated with human disease [21–24]. For clinical WGS, the sensitivity and coverage of CNVs have not been comprehensively investigated.

In this study, we performed a systematic analysis of the sensitivity and coverage of clinical WGS using 5 gold standard samples (NA12878, YH, NA24631, NA24694 and NA24695). Then, we applied clinical WGS for the reanalysis of 8 clinical cases with known disease-causing variants. The results may provide a reference for laboratories that perform clinical WGS.

## Methods

### Sequencing of samples

Based on the study design, DNA samples of NA12878, NA24631, NA24694 and NA24695 were procured from Coriell (Camden, NJ). One microgram of DNA was used for YH and the GIAB Chinese family to generate paired-end reads of 100 bp. The most widely used NA12878 was sequenced 2 times (WES and WGS) (Table 1). This study and all the protocols followed herein were approved by the ethics committee of BGI (NO. BGI-IRB19143).

**Table 1:** Sample information

Sample name	Sequencing	Platform	DNA input	PCR/PCR-free	Read length	Mean depth
NA12878-1	WGS	MGISEQ-2000	1 μg	PCR	PE100	197
NA12878-2	WES	MGISEQ-2000	300 ng	PCR	PE100	220
YH	WGS	MGISEQ-2000	1 μg	PCR	PE100	151
NA24631	WGS	MGISEQ-2000	1 μg	PCR	PE100	111
NA24694	WGS	MGISEQ-2000	1 μg	PCR	PE100	115
NA24695	WGS	MGISEQ-2000	1 μg	PCR	PE100	117

### Alignment and variant calling

In this study, a standard bioinformatics pipeline was used for analysis of all the samples (Additional files 1, 2). To eliminate variability due to differences among various bioinformatics tools, we developed a standard bioinformatics pipeline, which included current widely used tools (Additional files 1, 2). In general, after removal of sequencing adapters and low-quality reads, “clean reads” were aligned to the GRCh37 with BWA 0.7.12-r1039 [25]. Genome Analysis Toolkit (GATK)-package-4.0.11.0 [26] MarkDuplicates was used to remove duplicate reads. After realignment around indels and quality score re-calibration using GATK-package-4.0.11.0 [27], VCF files were generated using GATK for further analysis. For the CNV detection, 3 widely used tools (CNVnator [28], BreakDancer [29] and LUMPY [30]) were performed in this study.

After trimming sequencing adapters and consecutive low-quality bases using fastp [31], the clean reads of NA12878-1 were down-sampled by the sequencing depth of 10X (NA12878-1_10X), 20X (NA12878-1_20X), 30X (NA12878-1_30X), 40X (NA12878-1_40X), 50X (NA12878-1_50X), 60X NA12878-1_60X), 70X (NA12878-1_70X), 80X (NA12878-1_80X), 90X (NA12878-1_90X), 100X (NA12878-1_100X), 120X (NA12878-1_120X) and 150X (NA12878-1_150X) using seqtk (https://github.com/lh3/seqtk). The high-coverage NA12878-1 sample was sequenced with the MGISEQ-2000 platform to an average depth of ~ 197X (PE100) using PCR-based library construction. The clean reads of YH were also down-sampled by the sequencing depth.

### Sensitivity and positive predictive value of variant calls

To evaluate the performance of variant calls in detecting true genotypes, the high-confidence calls (SNPs and indels) for GIAB sample HG001 (NA12878) and HG005/HG006/HG007 (Chinese son/father/mother) were considered true-positive calls (v3.3.2) [32]. We restricted the calculation of sensitivity (high-confidence calls detected by our method/all high-confidence calls in GIAB), specificity (sites called as a reference using our method/all reference sites in GIAB), accuracy (percent of calling agreement of our method when compared with GIAB) and PPV (high-confidence calls detected by our method/all variants detected by our method) of variant calls to the high confidence region (v3.3.2) [32]. High-confidence variant calls and regions tend to include a subset of variants and regions that are easier to detect [32]. The sensitivity and PPV of variant calls in the YH sample were also evaluated. The alleles validated by the Illumina 1 M BeadChip were considered “true-positive” calls for YH [33]. Genotype quality (GQ) and DP were used to filter out variants with erroneous variant calls.

### Breadth of coverage for disease-associated genes and CNVs

To assess the recommended depth for proband-only WGS in clinical diagnostics, we collected a total of 6 gene sets for coverage analysis of disease-associated genes: ACMG59 [34], ClinVar [35] (3824 genes, accessed on 19 February 2019), Genetic Home Reference [36] (1471 genes, accessed on 2 July 2019), HGMD [37] (8171 genes, professional March 2018), OMIM [38] (3835 genes, accessed on 4 April 2018) and Orphanet [39] (2405 genes, accessed on 2 July 2019). For the annotation of gene regions, we used the information available in NCBI annotation release 104. For different transcripts, we first used the transcripts used in the HGMD database. The rest of the gene region consisted of a combination of the regions of all transcripts. Coverage analysis of the 12 down-sampling samples of NA12878-1 and YH for the 6 gene sets was performed for evaluation.

The genes in a single gene set of the 6 gene sets were incomplete. To generalize a new gene list containing all the putative disease-associated genes, we compiled a list of 8394 putative disease-associated genes from the 6 gene sets (Additional file 2: Table S9). This new gene set was generated using the following criteria: (1) a gene was retained when it was recorded with an association status of “assessed” in the Orphanet database and had one of the following association types: (a) “Disease-causing germline mutation(s) in”, (b) “Disease-causing germline mutation(s) (gain of function) in”, (c) “Disease-causing germline mutation(s) (loss of function) in”, (d) “Major susceptibility factor in”, (e) “Modifying germline mutation in”, (f) “Role in the phenotype of”, (g) “Candidate gene tested in”; (2) a gene was retained when it was recorded as a disease-related gene in the Genetic Home Reference database; (3) a gene was retained when it was recorded as a disease-related gene and the molecular basis of the disease was known, unless the inheritance of the disease was recorded as “SMu” (somatic mutation) only in the OMIM database; (4) a gene was retained when at least one variant of the gene was recorded as a probable/possible pathological mutation in the HGMD database; (5) a gene was retained when at least one variant of the gene was recorded as pathogenic or likely pathogenic in the ClinVar database; (6) a gene was retained unless it was not recorded with a well-defined gene ID or genome coordinate information in NCBI annotation release 104. This new gene list was used in the comparison of WES and WGS and is ideal for coverage analysis of clinical WGS.

We also performed coverage analysis of the 12 down-sampling samples of NA12878-1 and YH for CNVs described in the DECIPHER database (GRCh37_v9.29). Detailed information can be found in Additional file 2: Table S4.

### CNV analysis

Unlike SNPs and indels, there is no perfect "gold standard" CNV dataset for benchmarking. In this study, to assess the recommended depth for proband-only WGS in clinical diagnostics, we evaluated the sensitivity of CNV detection using 3 CNV call sets of NA12878 from published papers [40–42]. CNV call set 1 was also used by Haraksingh et al. [40] for the benchmarking of CNV detection from 17 commercially available arrays and low-coverage WGS. CNV call set 2 was determined by a machine learning-based approach (svclassify) and obtained from the GIAB Consortium benchmark SV calls resource [41]. CNV call set 3 included 874 deletions detected by both reference-based (a custom pipeline and PBHoney using both raw and error-corrected reads) and assembly-based analysis via single-molecule technologies [42]. All 3 CNV call sets have been previously compiled from NA12878 for benchmarking and downloaded for further analysis in the current study (Additional file 2: Table S10).

In the 12 down-sampling samples of NA12878-1, CNVnator (v0.3.2) [28], BreakDancer (v1.4.5) [29] and LUMPY (v0.2.13) [30] were assessed for CNV detection sensitivity with default or recommended parameters. In a CNV call set, true positives were classified as CNVs with at least a 50% reciprocal overlap with CNVs in the call set (BEDTools) [43]. For benchmarking, we determined the number of gold standard CNVs detected in the 12 down-sampling samples of NA12878-1 for the 3 CNV call sets.

### Sensitivity and PPV of variant calls in the Chinese trios

To test the sensitivity and PPV of variant calls for trio-based WGS, we investigated the sensitivity and PPV when taking advantage of the family-based trio information in the Chinese trios. Using the segregation pattern, we focused on the autosomes and X chromosome of NA24631, NA24694 and NA24695. Taking advantage of the family-based trio design, we analyzed all variants (DP ≥ 10X) of both parents, where one parent was consistently called as homozygous for the reference allele and the other as homozygous for the alt allele. We then used the variant calls (SNPs and indels) in the offspring to test the sensitivity and PPV for these loci.

## Results

### Study design

To test the sensitivity and coverage of clinical WGS, the Genome in a Bottle (GIAB) sample HG001 (NA12878), HG005 (NA24631)/HG006 (NA24694)/HG007 (NA24695) (known as the Chinese son/father/mother) and YH cell line (a human lymphoblastoid cell line from first Asian genome donor) [33] were collected and sequenced using the MGISEQ-2000 platform. All the samples used in this study are listed in Table 1.

**Fig. 1:**
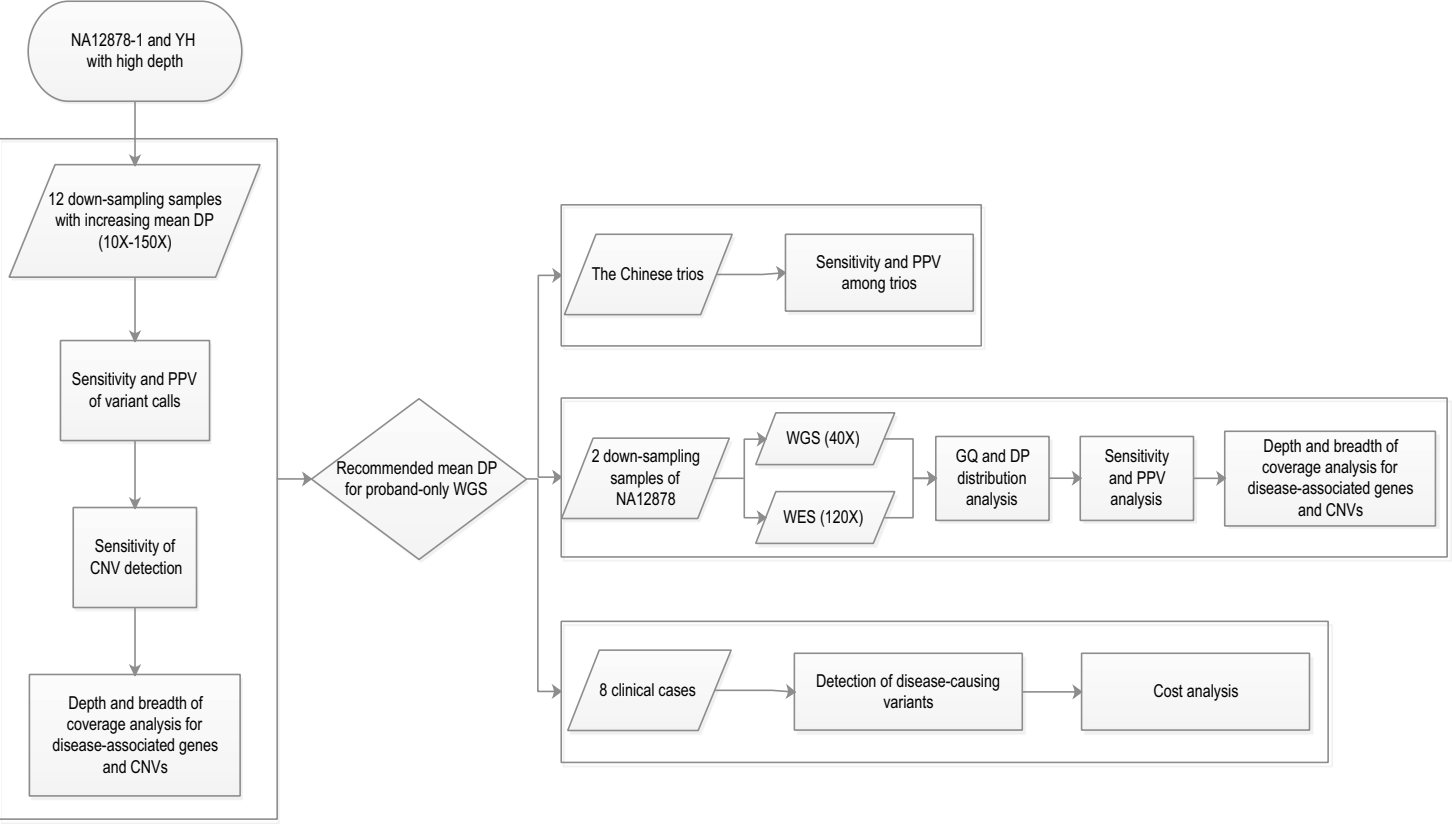
Study design

Figure 1 shows the overall design of this study. First, to assess the recommended depth for proband-only WGS in clinical diagnostics, the analysis of the sensitivity and positive predictive value (PPV) of high-confidence SNPs/indels, the sensitivity of CNV detection, and the depth and breadth of coverage for disease-associated genes and CNVs were performed using down-sampling samples of NA12878-1. Down-sampling samples were randomly down-sampled to a certain sequencing depth using seqtk (https://github.com/lh3/seqtk). The results of the sensitivity and PPV from a single genome may be difficult to generalize to a range of samples [44]. Consequently, in this part, similar analyses were also performed for down-sampling samples of another high depth sequencing sample of YH. After determining the recommended mean DP for the proband-only WGS, the Chinese trios (NA24631, NA24694 and NA24695) with the recommended mean DP were used to test the sensitivity and PPV of trio-based WGS when taking advantage of the family-based trio design in clinical WGS. Using down-sampling samples of NA12878-1 and NA12878-2, we also compared the performance of WES and WGS using the recommended mean DP. Finally, we analyzed 8 clinical cases with known disease-causing variants using our WGS pipeline (Fig. 1).

### Sensitivity, specificity, accuracy, and positive predictive value of variant calls

**Fig. 2:**
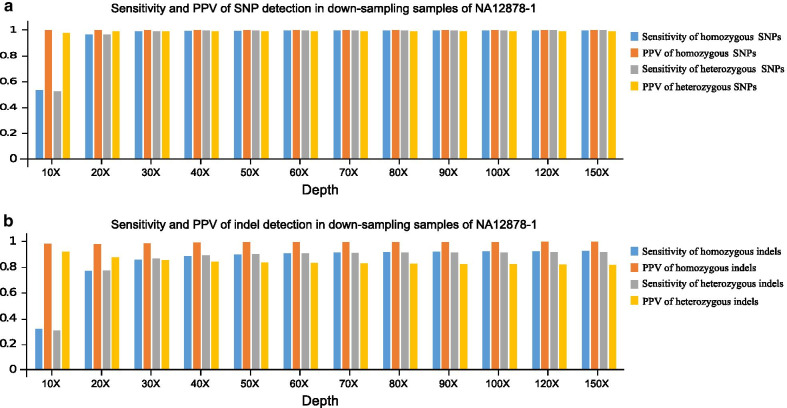
Sensitivity and PPV of variant calls from 12 down-sampling samples of NA12878-1. **a** Sensitivity and PPV of SNP detection in down-sampling samples of NA12878-1; **b** sensitivity and PPV of indel detection in down-sampling samples of NA12878-1

The mean DP was recognized as a general indicator of overall sensitivity for SNV/indel detection [4]. To reveal the sensitivity of proband-only WGS for SNP/indel detection, 12 down-sampling samples of NA12878-1 with an increasing mean DP (10X–150X) were evaluated. For the 12 down-sampling samples of NA12878-1, we restricted the calculation of sensitivity and PPV of SNPs/indels to the high confidence region (v3.3.2). GQ (≥ 20) and DP (≥ 10) were used to filter out variants with erroneous variant calls. As a result, when the mean depth is more than 40X, the sensitivity of detecting homozygous and heterozygous SNPs is more than 99.25% and 99.50%, the sensitivity of homozygous and heterozygous indels is more than 88.50% and 89.09%, respectively. We also restricted the calculation of specificity and accuracy of our method to the high confidence region. Both the overall specificity and accuracy of our method was more than 99.99% when the mean depth was more than 40X. The PPV (high confidence region) for homozygous and heterozygous SNPs exceeded 99.97% and 98.96%, and the PPV for homozygous and heterozygous indels exceeded 98.93% and 84.26%, respectively. Heterozygous indels showed the lowest PPV. Considering the sensitivity of SNP/indel detection and sequencing costs, a sequencing depth of ~ 40X provided the best value for SNP/indel detection, as indicated by the trends in the sensitivity results (Fig. 2).

The sensitivity of homozygous and heterozygous SNPs exceeded 96.48% and 96.59%, and it reached a plateau even with a mean depth of only 20X. The sensitivity significantly increased with sequencing depth from 10 to 30X for both homozygous and heterozygous indels, but it reached a plateau at an ~ 40X mean depth. A mean depth of 40X could provide a percentage of more than 99.05% for sites covering more than 20X in the high confidence region. However, clinical scientist should know that, even with a DP of ~ 150X, the sensitivity and PPV is still not 100%. With a DP of 150X, the sensitivity for homozygous and heterozygous SNPs was 99.70% and 99.81%, and the sensitivity of homozygous and heterozygous indels reached 92.57% and 91.57% respectively. With a DP of 150X, the PPV for homozygous and heterozygous SNPs was 99.97% and 98.82%, and the PPV for homozygous and heterozygous indels reached 99.61% and 81.42%, respectively.

The results from a single genome may be difficult to generalize to a range of samples [44]. Consequently, in this study, we also performed a sensitivity and PPV analysis of SNPs (in the 1 M validated region) for down-sampling samples of another high-depth sequencing sample of YH (Additional file 2: Table S1). In the 1 M validated region, the alleles were validated by the Illumina 1 M BeadChip [33], and similar results were obtained.

### Sensitivity of CNV detection

**Fig. 3:**
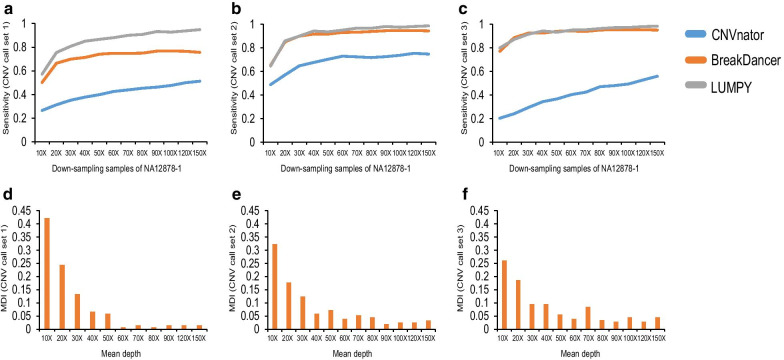
CNV detection in the 12 down-sampling samples of NA12878-1 using 3 CNV call sets. **a** Sensitivity of the 12 down-sampling samples of NA12878-1 for CNV call set 1; **b** sensitivity of the 12 down-sampling samples of NA12878-1 for CNV call set 2; **c** Sensitivity of the 12 down-sampling samples of NA12878-1 for CNV call set 3; **d** MDI value for CNV call set 1; **e** MDI value for CNV call set 2; **f** MDI value for CNV call set 3

To detect the sensitivity of proband-only WGS for CNV detection, the 12 down-sampling samples of NA12878-1 (10X–150X) with increasing mean DP were evaluated. CNVnator (read depth) [28], BreakDancer (read pair) [29] and LUMPY (read depth and read pair) [30] were used for the detection of CNVs for the 12 down-sampling samples. In this study, a total of 3 CNV call datasets were assessed. The overall sensitivity of CNVnator, BreakDancer and LUMPY of the 12 down-sampling samples for CNV call set 1 (BreakDancer only included the detection of deletions in CNV call set 1), CNV call set 2 and CNV call set 3 is shown in Fig. 3. In general, CNV calling is reliable with an increasing DP (Fig. 3a–c). At increasing sequencing depths, the trends of the sensitivity curves for the 3 CNV tools were different from one another. CNVnator showed a wide range of sensitivity with varying DP, and the sensitivity visibly increased with the mean depth, indicating that the sensitivity of CNV detection was positively correlated with the sequencing depth.

We also observed that the size of the CNVs might influence the sensitivity of CNV tools. The performance of each tool varied along with the size of CNVs (Additional file 1: Figs. S1-S9). Taking the deletions in CNV call set 1 as an example, the widely used tool CNVnator may not be suitable for CNV detection when the size of the CNV is less than 1 kb. When the CNV size was less than 1 kb, the sensitivity significantly increased with sequencing depth from 10 to 30X but reached a plateau at a depth of ~ 40X (Additional file 1: Figure S10). This result indicated that when the CNV size was less than 1 kb, the detection rate was greatly influenced by the sequencing depth, which was less obvious when the CNV size ranged between 6 and 70 kb. However, BreakDancer provided a better performance for deletion detection for all CNV sizes. These results suggested that clinical scientists should pay more attention to the selection of CNV tools when focusing on different CNV sizes.

The selection of CNV call set and CNV detection tools may influence the sensitivity of CNV detection, making the assessment of the recommended depth for CNV detection of proband-only WGS difficult. To investigate the minimum requirement of mean DP for CNV detection in proband-only WGS, using 3 CNV call sets (CNV call set 1, 2, 3) and the detection results of 3 CNV tools (CNVnator, BreakDancer and LUMPY), we defined a “miss detection index” (MDI) value in this study. The MDI value for a specific mean DP is defined as the frequency when the specific mean DP shows the “lowest” sensitivity for a different CNV size in a CNV call set. Without regard to selection of the CNV call set and CNV detection tools, MID can be used to evaluate the recommended depth for CNV detection of proband-only WGS.

In the formula, M indicates the number of times when mean depth i shows the “lowest” sensitivity of CNV detection, and N indicates the total number of times for all depths showing the “lowest” sensitivity of CNV detection. To obtain qualified CNV sizes in a CNV call set for evaluation, some criteria must be fulfilled for a CNV size (Additional files 1, 2). Detailed criteria and examples of the calculation of MDI can be found in the Additional files 1, 2.

As a result, the MDI value at a depth of 10X, 20X, 30X and 40X ranked first in the down-sampling samples of NA12878-1 (Fig. 3d–f). The 10X–40X accounted for more than 71.98% of the total depth. Taking together the sensitivity of detecting CNVs and sequencing costs, a sequencing depth of ~ 40X provided the best value for CNV detection, as indicated by the trends in the sensitivity curves (Fig. 3a–c).

### Depth and breadth of coverage for disease-associated genes and CNVs

Although WGS is better than WES for variation detection in patients with genetic disorders, the coverage of coding exons in key disease-associated genes of WGS has not been fully evaluated. To investigate the breadth of coverage of proband-only WGS for disease-associated genes, the breadth of coverage of 6 gene sets for the 12 down-sampling samples of NA12878-1 (10X-150X) and YH with increasing mean depth were evaluated. For each exon of the coding genes, we calculated the percent of exonic bases covered at more than 10X depth, which was reported to provide 95% sensitivity for heterozygous SNVs in WES [4]. None of the 12 down-sampling samples of NA12878-1 and YH covered 100% of the coding exons in the 6 gene sets except for the ACMG59 gene set (Additional file 2: Table S2). The results obtained for the down-sampling samples of NA12878-1 appeared slightly better than down-sampling samples of YH, probably because of the total sequencing depth of NA12878-1 (~ 197) and YH (~ 151). Across the 6 gene sets, a limited range of variation was found in the down-sampling samples when the mean depth was more than 40X (Additional file 2: Table S2).

**Fig. 4:**
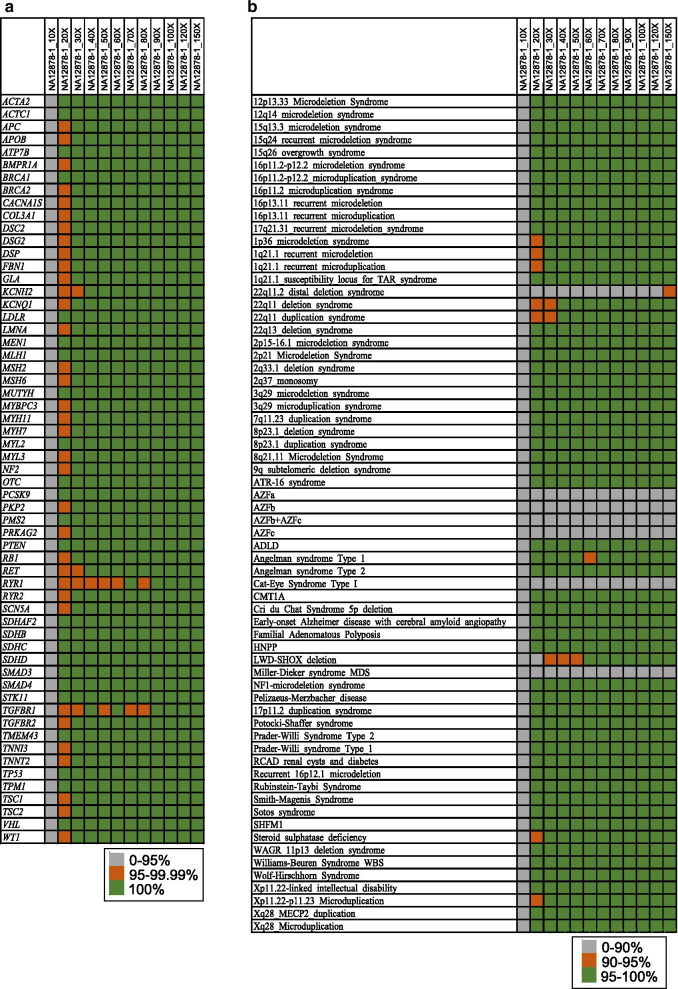
Depth and breadth of coverage for disease-associated genes and CNVs. **a** Depth and breadth of coverage for disease-associated genes; **b** depth and breadth of coverage for CNVs

Regarding the ACMG 59 genes, we also observed a range of variation in the breadth of coverage for the 12 down-sampling samples. Thus, a mean depth of more than 70X for YH and 90X for NA12878-1 covered 100% for all the ACMG 59 genes. A mean depth of 30X to 50X has been most widely used for WGS [19, 20]. The proportion of genes in the ACMG59 gene set covering 100% at ≥ 10X was 93.22%, 98.31% and 96.61% for NA12878-1_30X, NA12878-1_40X and NA12878-1_50X (with mean depths of ~ 30X, ~ 40X and ~ 50X). For a mean depth of ~ 30X, ~ 40X and ~ 50X for YH, we observed that 86.44%, 93.22% and 98.31% of the genes covered 100% at ≥ 10X. The breadths of coverage were significantly better when the average sequencing depth was more than 40X (Fig. 4a). The sites of all genes covered more than 99.9% when the sequencing depth was ~ 40X. Interestingly, we also observed poorly covered RYR1 and TGFBR1 genes in this study (Fig. 4a) in comparison to a previously published paper measuring the sensitivity and coverage of clinical WES [4], indicating that the poor coverage of RYR1 and TGFBR1 might be caused by the features of the gene regions and not the sequencing methods used. Clinical scientists must pay more attention to these genes when performing clinical WGS. Considering the cost of sequencing, a sequencing depth of ~ 40X provides the best value for the coverage of the ACMG 59 gene set, as indicated by the trends in breadth of coverage value of the ACMG 59 genes. We observed similar patterns for down-sampling samples of YH (Additional file 2: Table S3). We also examined the percentage of genes at ≥ 20X coverage in the ACMG 59 gene set, which could provide 99% sensitivity for heterozygous SNVs [4]. We found that 81.36% and 59.32% of the genes covered 100% for NA12878-1 and YH when the mean DP was ~ 40X.

CNVs are another major part of human genetic variation, which are often found to be associated with human diseases. For the CNVs in the DECIPHER database, most CNVs could be well covered (more than 95% coverage) at a depth > 10X when the sequencing depth was 40X-50X for NA12878-1 (Fig. 4b). Clinical scientists should pay more attention to 22q11.2 distal deletion syndrome, Cat-Eye Syndrome Type I and Miller-Dieker syndrome, which showed a coverage of 88.96%, 4.54% and 84.43% even with a sequencing depth of 100X. Similar patterns were also observed for down-sampling samples of YH (Additional file 2: Table S4).

### Sensitivity and PPV among trios

The purpose of the analysis of trios was to test the sensitivity and PPV when taking advantage of the family-based trio information in clinical WGS. Although the sequencing depth of the Chinese trio was more than 100, here we used down-sampling samples with a mean depth of 44.00X, 43.77X and 43.05X for NA24631, NA24694 and NA24695, respectively. We concentrated on the depth of ~ 40X because the ~ 40X depth is the most widely used and recommended depth for WGS, which is also consistent with some of our previous results, especially for CNV detection.

**Fig. 5:**
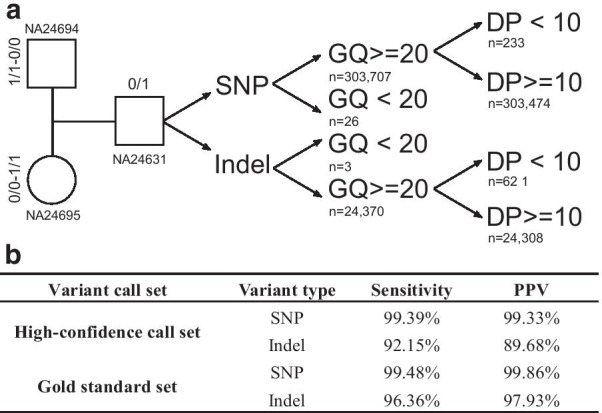
Variants analysis in the Chinese trios. **a** Variants used for trios analysis; **b** sensitivity and PPV of the “gold standard set” and the high-confidence call set for NA24631

In this study, we took advantage of the family-based trio design (Fig. 5a) to calculate the sensitivity and PPV in high confidence regions (NISTv3.3.2/GRCh37) [32]. The analysis was restricted to variants with DP ≥ 10X and GQ ≥ 20 (Fig. 5a). We focused on the loci where one parent was homozygous for the alt allele and the other was homozygous for the reference allele. For these variants, the offspring should be heterozygous, thus providing a new “gold standard set” for NA24631 (the offspring). In comparison to the high-confidence calls of NA24631 provided by NIST (the high-confidence call set), the new gold standard set could be used to test the sensitivity and PPV for trio-based WGS. Figure 5b shows the results of the sensitivity and PPV of the “gold standard set” and the high-confidence call set for NA24631. As a result, the sensitivity of the “gold standard set” for SNP and indel detection was > 99.48% and > 96.36%, respectively, and the PPVs were 99.86% and 97.93%. Trio-based analysis showed great improvement for the PPV of indel detection (Fig. 5b), and PPV for indel detection improved from 89.68% (using the high-confidence call set) to 97.93% (using the “gold standard set”).

### WGS and WES

Here we evaluated the performance of WES (MGIEasy Exome FS Library Prep Set) and WGS (MGIEasy PCR-Free DNA Library Prep Set) using DNA samples of NA12878. NA12878-1_40X and NA12878-2_120X for the evaluation. NA12878-1_40X was the down-sampling sample of NA12878-1 with a mean DP of 40X. NA12878-2_120X was a down-sampling sample of NA12878-2 with a mean DP of 120X, which is typical and the current standard for clinical WES [45]. Two quality parameters for variation detection (DP and GQ), sensitivity for SNV/indel detection, and the breadth of coverage of the list of 8394 putative disease-associated genes and CNVs were compared in this section.

DP and GQ are two main parameters assessing the quality of variant calls, which are often used to filter out variants with erroneous variant calls [27, 33]. First, we investigated the GQ and DP distribution of NA12878-1_40X and NA12878-2_120X in the regions of the human genome covered by WES (59,082,036 bp). We found similar results to those obtained in a previous WES study [10]. The distribution of DP for the variants had a wider range in NA12878-2_120X than in NA12878-1_40X (Additional file 1: Figure S11), with a median depth of 94X but a mode at 63X and 58X for SNPs and indels, respectively, indicating low levels of coverage for a substantial proportion of variants. In contrast, the distribution of DP was nearly normal for NA12878-1_40X, with a median at 42X and coinciding mode at 41X for both SNPs and indels (Additional file 1: Figure S11). The vast majority of variants called by NA12878-2_120X had a GQ close to 100 and fluctuated along with the GQ scores. The distribution of GQ for variants in NA12878-1_40X showed a mode in low GQ area (with a peak value close to GQ 41), which was probably caused by insufficient variant calls in the WES regions.

For the detection of SNVs and indels, “true positive” calls were further restricted to the regions of the human genome covered by WES (59,082,036 bp). A total of 44,726 variants were used for evaluation. DP and GQ filtering were not used in this part. In general, the sensitivity and PPV of WGS (NA12878-1_40X) were higher than WES (NA12878-2_120X) for the 44,726 variants (Additional file 2: Table S5), except for the PPV for homozygous indel detection. For 90.39% of the gold SNPs, WES and WGS yielded the same genotype. More than 63.41% of these concordant SNVs were identified as heterozygous, which was similar to those obtained in previous WES studies [4, 20, 46].

Then, we investigated the breadth of coverage of NA12878-1_40X and NA12878-2_120X in the 8394 putative disease-associated genes and CNVs (DECIPHER database). As a result, WGS showed better coverage of both putative disease-associated genes and CNVs. More than 99.77% of the exon region sites of the 8394 putative disease-associated genes were covered with a depth ≥ 10 for NA12878-1_40X, while NA12878-2_120X covered 99.46% of the exon regions. NA12878-2_120X was poorly covered for CNVs in the DECIPHER database (Additional file 2: Table S6). More than 69.69% of the CNVs showed a coverage less than 10%.

### Analysis of 8 clinical cases with known disease-causing variants

In this study, samples of 8 clinical cases with known variants of various types were recruited and reanalyzed using our WGS pipeline. All 12 variants (Additional file 2: Table S7) were validated previously by methods other than MPS technology, including 8 SNVs, 3 indels and 1 CNV. Seven and five variants were classified as pathogenic and likely pathogenic, respectively, according to the ACMG guidelines for variant classification [22, 23].

Focusing on SNVs, indels and CNVs, we applied our method to the 8 clinical cases using singleton WGS. Variants were manually assessed for quality and interpreted according to the American College of Medical Genetics and Genomics (ACMG) guidelines for variant classification [22, 23]. All the previously validated variants (Additional file 2: Table S7) were successfully detected using our WGS pipeline, which further demonstrated the sensitivity of the method.

Based on the sequencing DP, the average cost for the WGS approach was ~ $490 per sample for the 8 clinical cases in the current study. The overall cost (from DNA extraction to reporting) for a single case with a mean sequencing depth of ~ 40X was approximately $600, including ~ $280 for chemicals (DNA extraction, library construction and sequencing), ~ $220 for labor, and ~ $100 for depreciation expenses.

## Discussion

Thus far, more than 8000 Mendelian diseases have been recorded by OMIM (Online Mendelian Inheritance in Man), more than 5000 of which have a phenotype description and molecular basis. The rapid development of massively parallel sequencing (MPS) technology has revolutionized the field of genetic diagnosis in the clinical setting, making it possible for MPS to be a routine part of clinical care. The emergence of MPS technology makes multigene sequencing, exome level sequencing, and even genome level sequencing possible, which have been increasingly widely used in clinical diagnoses for genetic diseases. In this study, we performed a comprehensive analysis of the sensitivity and coverage of clinical WGS as a diagnostic test for genetic disorders. First, we analyzed the sensitivity and PPV value of high-confidence SNPs/indels, as well as the sensitivity of CNV detection, using down-sampled NA12878 and YH. A new MDI value was defined for the evaluation of CNV detection; then, we investigated the depth and breadth of coverage for disease-associated genes and CNVs in down-sampling samples of NA12878 and YH. A new gene set and a CNV call set were generated during this process. Next, we compared the performance of WES and WGS for DNA samples of NA12878. We also tested the sensitivity and PPV of variant calls when taking advantage of the family-based trio design in clinical WGS. Finally, we analyzed 8 clinical cases with known disease-causing variants using our WGS pipeline. The results suggested that WGS can be used in the detection of SNVs/indels/CNVs with high sensitivity, and the current standard of a mean depth of ~ 40X may be a cost-effective sequencing depth for SNV/indel detection and the identification of most CNVs. WGS is likely to be widely used and become a routine part of clinical care in the near future.

Although a mean depth of 40X was recommended for clinical WGS in this study, certain disease-causing variants may be detected with low depth (Additional file 2: Table S7, 3 out of 12 variants with 17X). In real clinical setting, these variants will be caught and should be further validated using methods other than MPS. Using the 12 down-sampling samples of NA12878-1, we found that a mean depth of 40X could provide a percentage of more than 99.05% for sites covering more than 20X in the high confidence region, which means that, even reaching a mean depth of 40X, there are still sites with a depth of less than 20X. Variants called at these sites with low depth may be false positives, however, they will still be caught. Further interpretation of these variants should be conducted combined with the phenotype of the patient. If disease related or known diseasing causing mutations are found with low depth, further validation is recommended to be conducted using methods other than MPS.

During the analysis of the PPV of indels in the high confidence regions, we detected a slight decline for heterozygous indels in the 12 down-sampling samples of NA12878 with the increase in DP. One reasonable explanation is that with the increase in depth, both true positives and false positives increased. We found, however, that the false positives increased a little bit faster than the true positives, and as a result, PPV declined. A massive scale p population-based polymorphism database and further filtering of the variants may be useful to solve this problem.

In addition to SNVs and indels, another major part of human genetic variation is copy number variation. According to clinical requirements, choosing suitable methods and tools for accurate and reliable detection of CNVs is important for clinical diagnostics. WGS can detect nearly all known genetic variations. However, our results indicated that, although CNV calling was reliable with increasing DP, the performance of CNV tools varied immensely. Finding the right tool for CNV detection is difficult for clinical scientists. Our results suggested that read pair methods (BreakDancer in particular) showed the best performance for the identification of deletions of more than 1 kb. Moreover, although some “gold standard” CNV call set has been widely used in published papers [40–42], with the lack of validation of various methods, some CNVs may be false positives with inaccurate or low-resolution boundaries. Factors (such as CNV size and the selected “gold standard” set) may also influence the sensitivity of CNV detection, making it difficult to determine the sufficient DP for CNV detection. In this study, we introduced the concept of MDI to solve this problem. The MDI value for a specific mean DP is defined as the frequency when the specific mean DP shows the “lowest” sensitivity for different CNV size in a CNV call set, which was defined to evaluate the recommended depth for CNV detection. Finally, we found that the current standard of a mean depth of 40X might be sufficient for the identification of most CNVs. Based on the results already obtained, MDI can be used to reflect the performance of CNV detection for certain CNV call sets and CNV tools. However, there are limitations of our analysis. First, MDI has not been validated elsewhere. Based on the definition of MDI, we can see that MDI is an objective value for the assessment of recommended depth for CNV detection. Lacking validation may block the application of MDI. Second, we only sampled a small number of CNV callers (CNVnator, BreakDancer and LUMPY). In a real clinical setting, the application of more than one CNV calling algorithm should be considered to improve the sensitivity of CNV detection. Analysis of the sensitivity of CNV detection with all available CNV tools would be an interesting research topic. In addition, we found that additional coverage is associated with an overall increase of the sensitivity for CNV detection; however, this is less obvious as the CNV size is more than 100 kb, as described in another published paper [26].

There is no perfect "gold standard" CNV dataset for benchmarking. Thus, in this study we compiled a list of 2022 “likely true positives” from the 3 most commonly used CNV call sets of NA12878 from published papers [40–42] for benchmarking (Additional file 2: Table S8), including 1912 deletions and 110 duplications. This new set represents a combination of the 3 CNV call sets after evaluation. For this new CNV call set, we defined true positives as the variants detected by at least one CNV tool (CNVnator, BreakDancer and LUMPY) with more than 50% reciprocal overlap and confirmed by visualization of the copy ratio using an in-house script. This new set is an ideal “gold standard” CNV call set of NA12878 for clinical WGS benchmarking.

When comparing the GQ and DP distribution of NA12878-1_40X (WGS) and NA12878-2_120X (WES), the regions were restricted to the human genome covered by WES (59,082,036 bp). In this region, NA12878-2_120X detected 54,290 SNPs and 7918 indels, while NA12878-1_40X identified 53,107 SNPs and 6577 indels. High depth based WES method (NA12878-2_120X) detected more SNPs and indels than WGS based method (NA12878-1_40X). As is shown in Additional file 1: Figure S11, low levels of coverage for a substantial proportion of variants were detected in NA12878-2_120X. Higher sensitivity for WGS (NA12878-1_40X) were also detected for the 44,726 variants (Additional file 2: Table S5). One reasonable explanation for the different "variation count" in NA12878-1_40X (WGS) and NA12878-2_120X (WES) is that, in the sites with low levels of coverage in NA12878-2_120X, more false positives were called.

The turn-around time and cost of WGS are two key points for the clinical application of WGS. The entire workflow of this method lasts approximately 11–12 days from the recruitment of sample to clinical reporting for one sample. BGI produced MegaBOLT (MegaBOLT bioinformatics analysis accelerator) along with the sequence platform MGISEQ-2000, which is an MGI self-developed and MPS-concentrated hardware accelerating system for bioinformatics analysis. MegaBOLT supports the analysis of WGS and WES, and it is 20 times faster than the traditional GATK approach, which can be used to shorten the bioinformatics process. Along with the development of automated diagnostic tools [47, 48], which could be used to prioritize patient phenotypes and expedite genetic disease diagnosis, the turn-around time of WGS could be further reduced. The overall cost, including chemicals, labor, and depreciation expenses for the WGS approach was $600 per sample (~ 40X depth). Sequencing accounted for nearly half of the total cost. In general, variant calling is more reliable with increasing DP. However, there is a detection ceiling for some genes and/or regions (such as regions related to Miller-Dieker syndrome), which cannot be solved by increasing the sequencing depth. The cost and sensitivity of WGS must be balanced. Our results suggest that the current standard of a mean depth of 40X may be sufficient for the identification of most SNVs and CNVs. Reduction of the cost and turn-around time would further improve the clinical application of WGS.

## Conclusions

In summary, the successful application of WGS as a diagnostic test for genetic disorders in the real clinical setting requires a comprehensive assessment of the depth and breadth of coverage and the sensitivity of WGS. In this study, we observed variation in the detection of SNV/indel/CNV and substantial variation in the coverage of medically implicated genes and CNVs. In the real clinical setting, it would be advisable for clinical scientists to determine the range of sensitivity and PPV for different classes of variants for a particular WGS pipeline, which would be useful when interpreting and delivering clinical reports. We believe that WGS is likely to change the clinical diagnosis of rare and undiagnosed diseases in the near future.

## Supplementary Information

**Additional file 1**. Supplementary Tables.

**Additional file 2**. Supplementary Material.

## Data Availability

The datasets of the GIAB samples used and analyzed during the current study have been deposited in the CNSA (https://db.cngb.org/cnsa/) of CNGBdb with accession code CNP0000813. The data of the 8 clinical cases generated and analyzed during the current study is not publicly available as they are patient samples and sharing them could compromise research participant privacy. Other databases used in this study are listed below: GRCh37: http://hgdownload.soe.ucsc.edu/goldenPath/hg19/chromosomes/. High-confidence calls (SNPs and indels) for GIAB sample HG001 (NA12878) (v3.3.2): ftp://ftp-trace.ncbi.nlm.nih.gov/giab/ftp/release/NA12878_HG001/NISTv3.3.2/GRCh37/. High-confidence calls (SNPs and indels) for GIAB sample HG005 (NA24631) (v3.3.2): ftp://ftp-trace.ncbi.nlm.nih.gov/giab/ftp/release/ChineseTrio/HG005_NA24631_son/NISTv3.3.2/GRCh37/. High-confidence calls (SNPs and indels) for GIAB sample HG006 (NA24694) (v3.3.2): ftp://ftp-trace.ncbi.nlm.nih.gov/giab/ftp/release/ChineseTrio/HG006_NA24694_father/NISTv3.3.2/GRCh37/. High-confidence calls (SNPs and indels) for GIAB sample HG007 (NA24695) (v3.3.2): ftp://ftp-trace.ncbi.nlm.nih.gov/giab/ftp/release/ChineseTrio/HG007_NA24695_mother/NISTv3.3.2/GRCh37/. ClinVar: https://www.ncbi.nlm.nih.gov/clinvar/. Genetic Home Reference: https://medlineplus.gov/genetics/. HGMD: http://www.hgmd.cf.ac.uk/ac/index.php. OMIM: https://omim.org/. Orphanet: https://www.orpha.net/. DECIPHER: https://decipher.sanger.ac.uk/.

## Abbreviations

WGS: Whole-genome sequencing
WES: Whole-exon sequencing
OMIM: Online Mendelian Inheritance in Man
MPS: Massively parallel sequencing
SNV: Single-nucleotide variant
Indels: Small insertions and deletions
CNV: Copy-number variant
ACMG: The American College of Medical Genetics and Genomics
GIAB: Genome in a Bottle
PPV: Positive predictive value
MDI: Miss detection index
DP: Depth of coverage
GQ: Genotype quality
GATK: Genome analysis toolkit
