# Supplementary material for: Characterizing sensitivity and coverage of clinical WGS as a diagnostic test for genetic disorders
Source: BMC Med Genomics. 2021 Apr 13;14:102. doi: 10.1186/s12920-021-00948-5 (PMC8045368; doi:10.1186/s12920-021-00948-5)

Characterizing sensitivity and coverage of clinical WGS as a diagnostic test for genetic disorders

Running title: sensitivity and coverage of clinical WGS

Authors: Yan Sun[1], Fengxia Liu[2,3], Chunna Fan[2,3], Yaoshen Wang[2,3], Lijie Song[2,3], Zhonghai Fang[2,3], Rui Han[2,3], Zhonghua Wang[2,3], Xiaodan Wang[2,3], Ziying Yang[2,3], Zhenpeng Xu[1], Jiguang Peng[1], Chaonan Shi[2,3], Hongyun Zhang[1], Wei Dong[4], Hui Huang[1], Yun Li[1], Yanqun Le[2], Jun Sun[2,3*] and Zhiyu Peng[1*]

[1]BGI Genomics, BGI-Shenzhen, Shenzhen, 518083, China

[2]Tianjin Medical Laboratory, BGI-Tianjin, BGI-Shenzhen, Tianjin 300308, China

[3]Binhai Genomics Institute, BGI-Tianjin, BGI-Shenzhen, Tianjin 300308, China

[4]BGI-Beijing Clinical Laboratories, BGI-Shenzhen, Beijing 100000, China

[*]Correspondence: Jun Sun (sunjun@bgi.com); Zhiyu Peng (pengzhiyu@bgi.com).

Yan Sun, Fengxia Liu, Chunna Fan and Yaoshen Wang contributed equally to this work.

Supplementary material

Bioinformatics pipeline

1. filter

1) fastp.V*_L*.sh: Filtering short reads

```
fastp --thread 10 –I [fastq file for the first of read pairs] –I [fastq file for the second of read pairs] –o [cleaned fastq file for the first of read pairs] –O [cleaned fastq file for the second of read pairs] -s 8 –j [statastics file in json format ] –h [statastics file in html format ]
```

2. align

1) align

Alignment with clean reads

```
bwa mem -M -t 8 –R "@RG\tID:sample id\tSM:sample id\tPL:platform" [GRCh37 reference] [cleaned fastq file for the first of read pairs]  [cleaned fastq file for the second of read pairs] | samtools view -S -b –o [output BAM file] -
```

2) sort

```
sambamba sort --memory-limit 23G -l 1 -t 8 --tmpdir=[temporary output directory] –o [output aligned BAM] [input BAM]
```

3) merge

```
samtools merge -R [chromosome tag] -c -p –f [output merged bam] [all bams of parts reads aligned ]
```

4) dup

```
java -Xmx20G -XX:ParallelGCThreads=2 \
-Djava.io.tmpdir=[temporary directory] –jar MarkDuplicates.jar INPUT=[each chromosome bam] OUTPUT=[output bam file] METRICS_FILE=[statistical file]
VALIDATION_STRINGENCY=SILENT
MAX_FILE_HANDLES_FOR_READ_ENDS_MAP=8000
```

5) fix

```
java -Xmx20G -Djava.io.tmpdir=[temporary directory] –jar gatk.jar FixMateInformation --VALIDATION_STRINGENCY SILENT –I [input bam] -O [output bam]
```

3. bam_chr

1) chr21.bqsr.sh

```
java -jar gatk.jar BaseRecalibrator –R [GRCh37 reference] –I [input bam] --tmp-dir [temporary directory] --known-sites [1000G vcf file] --known-sites [1000genome snp vcf file] --known-sites [dbSNP vcf file] --known-sites [1000genome snp vcf file] –O [1000genome snp vcf file]

java -Djava.io.tmpdir=[tmp directory] –jar gatk.jar ApplyBQSR –I [input bam] –bqsr [bqsr mediate file] –O [output bam file]
```

4. QC

1) bam split

```
Sambamba view -f bam -h -o [output bam] -L [bed] [input bam]
```

2) partial bam qc

```
bamdst -p [bed] -o [output directory] [input bam]
```

3) QC collection

```
Python wgs.qc_collect.py –filter [filter directory] –bamqc [ partial bam qc ] -o [output directory] --sex [sex tag] > [output file]
```

5. variant

1) variant calling

```
java -Xmx4G -XX:ParallelGCThreads=4 –jar gatk.jar HaplotypeCaller --tmp-dir tmp -ERC GVCF --correct-overlapping-quality true -A BaseQuality -A MappingQuality -A QualByDepth -A MappingQualityRankSumTest -A ReadPosRankSumTest -A FisherStrand -A StrandOddsRatio -A InbreedingCoeff –R
```

```
[reference file] –L [bed] –I [input bam] –O [output gvcf file]

java -Xmx4G -XX:ParallelGCThreads=4 –jar gatk.jar GenotypeGVCFs --tmp-dir
tmp –R [reference file] –V [gvcf file] –L [bed] –O [vcf file]
```

2) variant collection

```
bcftools concat -a -D -q 30 -O z –o [output vcf file] –f [vcf list file]

tabix -p vcf [input vcf]
```

3) snp vqsr

```
java –jar gatk.jar SelectVariants --tmp-dir=snp_javatmp –R [reference file] –variant
[vcf file] –O [snp vcf file] -select-type SNP

java –jar gatk.jar VariantRecalibrator --tmp-dir=snp_javatmp –R [reference file] –V
[snp vcf file] --resource
hapmap,known=false,training=true,truth=true,prior=15.0:hapmap_3.3.hg19.sites.vc
f.gz --resource omni,known=false,training=true,truth=false,prior=12.0:
1000G_omni2.5.hg19.sites.vcf.gz --resource
1000G,known=false,training=true,truth=false,prior=10.0:
1000G_phase1.snps.high_confidence.hg19.sites.vcf.gz --resource
dbsnp,known=true,training=false,truth=false,prior=2.0:dbsnp_138.hg19.vcf.gz -an
DP -an QD -an MQ -an MQRankSum -an ReadPosRankSum -an FS -an SOR -mode
SNP –O [temporary vqsr file of snp vcf ] --tranches-file [snp tranches vcf file]

java –jar gatk-package-4.0.11.0-local.jar ApplyVQSR --tmp-dir=[tmp directory] –R
[reference file] –V [snp vcf file] –O [output snp vcf file] --truth-sensitivity-filter-
level 99.0 --tranches-file [snp tranches vcf file] --recal-file [temporary vqsr file of
snp vcf ] -mode SNP

java –jar gatk.jar SortVcf –I [filtered snp vcf file] –O [output vcf file]
```

4) indel vqsr

```
java –jar gatk.jar SelectVariants --tmp-dir=[temporary directory] –R [reference file] –[input vcf file] –O [output snp vcf file] -select-type INDEL

java –jar gatk.jar VariantRecalibrator --tmp-dir= indel_javatmp –R [reference file] –V [indel vcf file]-resource mills,known=true,training=true,truth=true,prior=12.0: Mills_and_1000G_gold_standard.indels.hg19.sites.vcf -an DP -an QD -an MQ -an MQRankSum -an ReadPosRankSum -an FS -an SOR -mode INDEL –O [temporary vqsr file of indel vcf] --tranches-file [indel tranches vcf file]

java –jar gatk.jar ApplyVQSR --tmp-dir= [temporary directory] –R [reference file] –V [indel vcf file] –O [output indel vcf file] --truth-sensitivity-filter-level 99.0 --tranches-file [indel tranches vcf file] --recal-file [temporary vqsr file of indel vcf ] -mode INDEL

java –jar gatk.jar SortVcf –I [filtered indel vcf file] –O [output vcf file]
```

5) snp indel concat

```
java –jar gatk.jar MergeVcfs –I [input snp vcf file] –I [input indel vcf file] –O [output vcf file]

tabix -p vcf –f [input vcf file ]
```

6. SV

1) cnvNator

```
sh cnvnator_jiyinku.sh [chr tag] [output root file] [reference file directory] [output file]
```

2) BreakDancer

```
Perl bam2cfg.pl -v 20 bam >[output config file]
breakdancer_max -a [input config file] >[output ctx file]
```

```
perl filt_sv.pl -m 100 -x 1000000 -s 30 -d 5 -I [input ctx file] -o [output filter ctx]
```

3) ExonDepth

```
Perl run_SMN_CNV_control_v2.pl [bam list file] [bed file] [input gene file] [output file]
```

4) lumpy

Extract the discordant paired-end alignments

```
samtools view -b -F 1294 [input bam] |>[output bam]
```

Extract the split-read alignments

```
samtools view -b -F 1294 [input bam] extractSplitReads_BwaMem -i stdin|samtools view -Sb - >[output bam]
```

```
lumpyexpress -B [input bam] -S [split bam] -D [disco bam] -T [temporary directory] -x [bed] -o [output vcf file]
```

5) SMA

```
Sh all.sh [sample tag] [input bam] [sex] [output directory]
```

7. annotation

chr*_part*.sh

```
perl anno.pl [input vcf file] -t vcf -n 9 -b 1000 -q -g [male/female] –o [output file]


perl anno.acmg.pl [input file] > [output file]
perl update.Function.pl [input file] > [output file]
anno2xlsx –snv [input file] -redis -redisAddr 10.2.1.4:6380 -wgs
```

Miss detection index (MDI)

Unlike SNP and indel detection, CNV detection is more complicated. First, there are no perfect “gold standard” CNV dataset for benchmarking. Although some “gold standard” CNV call set is widely used in published papers, lacking validation of various methods, some CNVs may be false positives with wrong or low resolution boundaries; Second, our results showed that CNV size may influence the sensitivity of CNV detection (Supplementary Figure 1-9); Third, we also observed a substantial variation in the sensitivity of CNV detection across different tools. All these things make the assessment of the recommended depth for CNV detection difficult in proband-only WGS. In this study, we introduced the concept of MDI to solve these problems.

Using 3 CNV call sets (CNV call set 1, 2, 3) and the detection results of 3 CNV tools (CNVnator, BreakDancer and LUMPY), we defined a MDI value in this study. The MDI value for a specific mean DP is defined as the frequency when the specific mean DP shows the “lowest” sensitivity for different CNV size in a CNV call set. Without regard to selection of CNV call set and CNV detection tool, MID can be used to evaluate the recommended depth for CNV detection of proband-only WGS.

$$MDI_i = \frac{M_i}{N}$$

In the formula, M means the number of times when mean depth i shows the “lowest” sensitivity of CNV detection, N means the total number of times for all the depth that shows the “lowest” sensitivity of CNV detection. To obtain qualified CNV sizes in a CNV call set for evaluation, some criteria need to be fulfilled for a CNV size. Here, a CNV size is recognized unqualified if: 1) the number of detection rate for a specific mean depth (the detection rate is >10% less than the highest detection rate in this specific CNV size) is less than 3, this CNV size would be deleted because mean depth showed little influence on the detection rate. The “lowest” detection rate includes the lowest 3 detection rate; 2) the percentage of 0 detection rate of a CNV size is more than 50%; 3) the percentage of the same detection rate for a specific mean depth is more

than 95%. Taking CNV size into consideration, MDI value could mediate the influence of CNV tools. What is more, although some “gold standard” CNV set is widely used in published papers, lacking validation of various methods, some CNVs may be false positives with wrong or low resolution boundaries. MDI took the top sensitivity of different CNV tools as the detection ceiling, which did not require that all the CNVs in the dataset are “true positives”.

Here is an example of how to calculate the MDI value for a DP of 10X (Table S1). Table S1 shows the sensitivity of CNV detection in a virtual CNV call set using down-sampling samples with increasing mean DP (10X-100X). There are 3 CNV sizes (100-200bp, 201-300bp and 301-400bp) in this virtual CNV call set. Each CNV size contains 10 CNVs. For CNV size 1, the “lowest” sensitivities are 10% (10X), 20% (20X) and 30% (30X), so $M_{10X} = 1$, $M_{20X} = 1$, $M_{30X} = 1$. According to criteria 2 for a CNV size, CNV size 2 is unqualified because the percentage of 0 detection rate for this CNV size is more than 50% (7/10). For CNV size 3, the “lowest” sensitivities are 10% (10X), 20% (20X) and 30% (40X). Now $M_{10X} = 2$, $M_{20X} = 2$, $M_{30X} = 1$, $M_{40X} = 1$, N = 6 (the total number of times for all the depth that shows the “lowest” sensitivity of CNV detection). As a result, $MDI_{10X} = 2/6 = 0.33$, $MDI_{30X} = 1/6 = 0.167$.

Table S1

CNV size 1: 101-200bp (10 CNVs)										
DP	10X	20X	**30X**	**40X**	50X	60X	70X	80X	90X	100X
Sensitivity	10%	20%	**30%**	**80%**	80%	80%	90%	90%	90%	100%

CNV size 2: 201-300bp (10 CNVs)										
DP	10X	20X	30X	40X	50X	60X	70X	80X	90X	100X
Sensitivity	**0%**	**0%**	**0%**	**0%**	**0%**	**0%**	**0%**	50%	80%	90%

CNV size 3: 301-400bp (10 CNVs)										
DP	10X	20X	**40X**	**30X**	50X	60X	70X	80X	90X	100X
Sensitivity	10%	20%	**30%**	**40%**	40%	80%	90%	90%	90%	100%

Here is one example of how to calculate the MDI for a specific DP. The differences of MDI used in this example and in the main article are that it used 3 CNV call sets (CNV call set 1, 2, 3) and the detection results of 3 CNV tools (CNVnator, BreakDancer

and LUMPY) for calculation in the main article.

Supplementary Figure 1 Sensitivity of CNV detection in down-sampling samples of NA12878-1 by CNVnator (CNV call set 1)

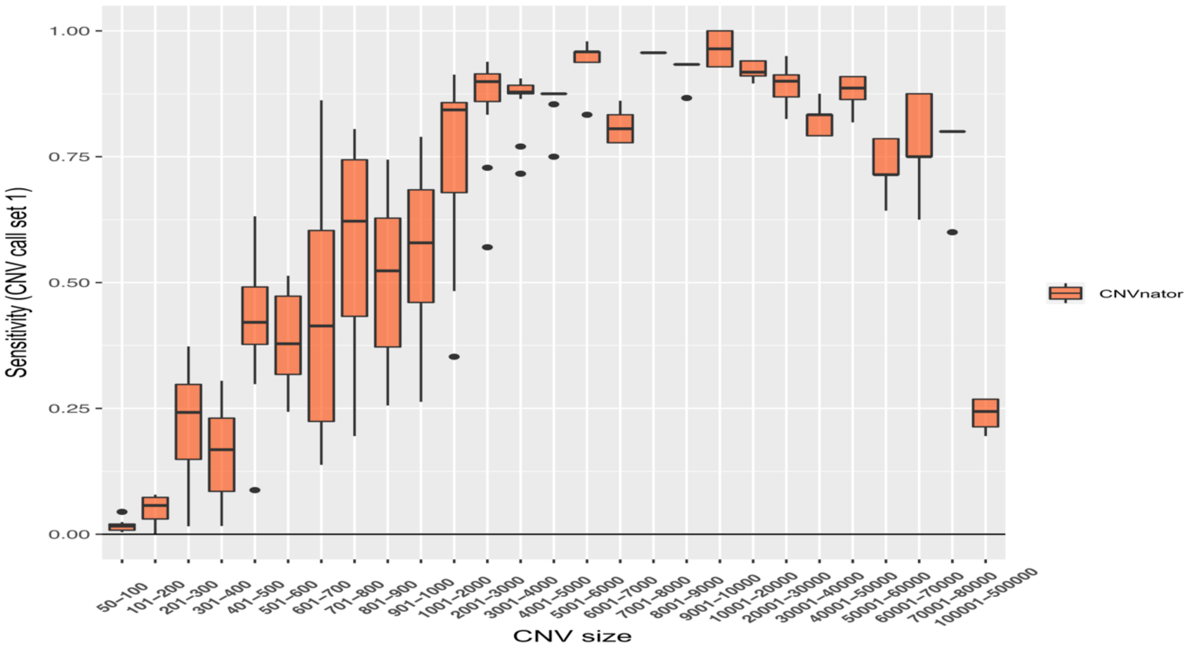

Supplementary Figure 2 Sensitivity of CNV detection in down-sampling samples of NA12878-1 by BreakDancer (CNV call set 1)

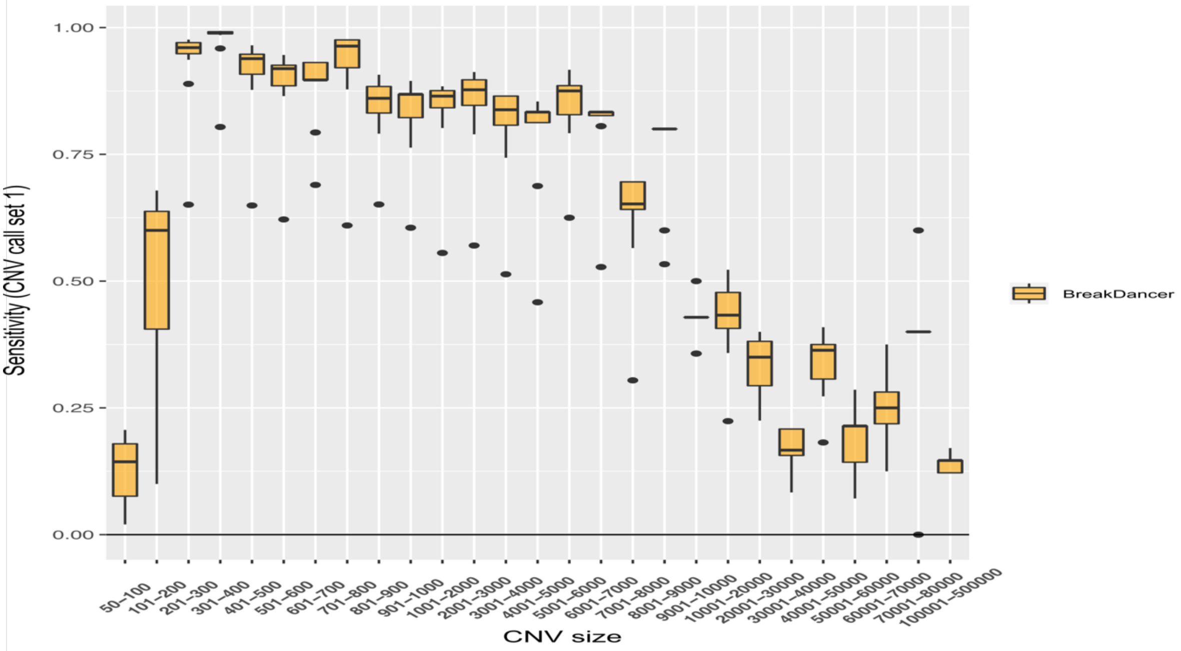

Supplementary Figure 3 Sensitivity of CNV detection in down-sampling samples of NA12878-1 by LUMPY (CNV call set 1)

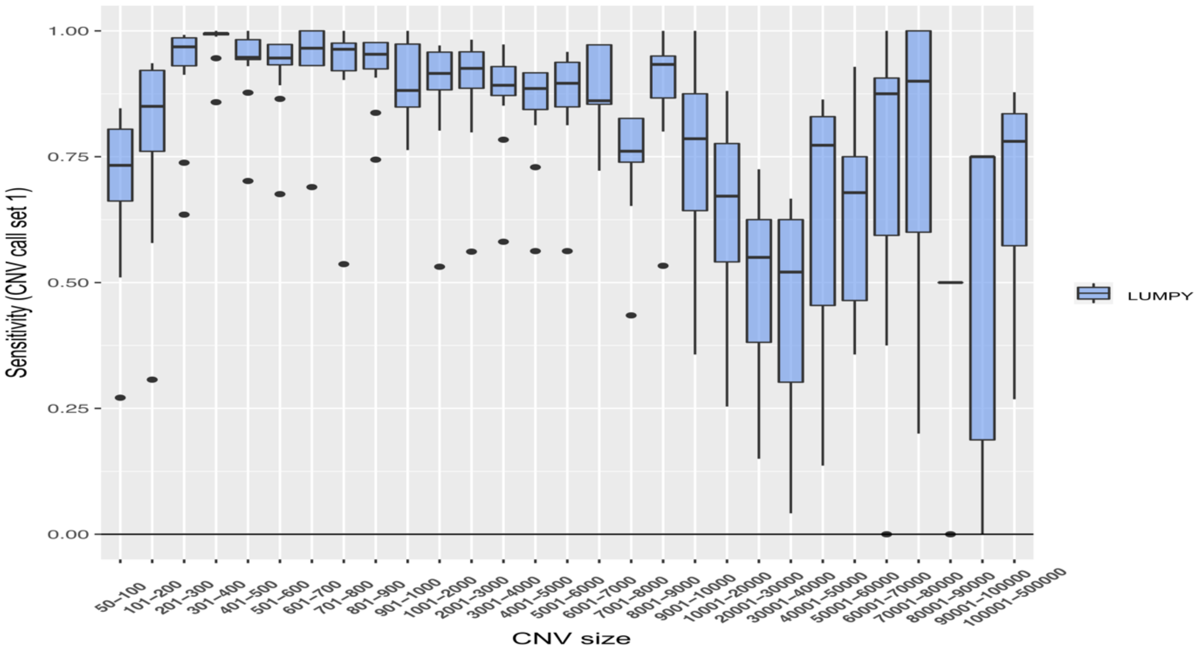

Supplementary Figure 4 Sensitivity of CNV detection in down-sampling samples of NA12878-1 by CNVnator (CNV call set 2)

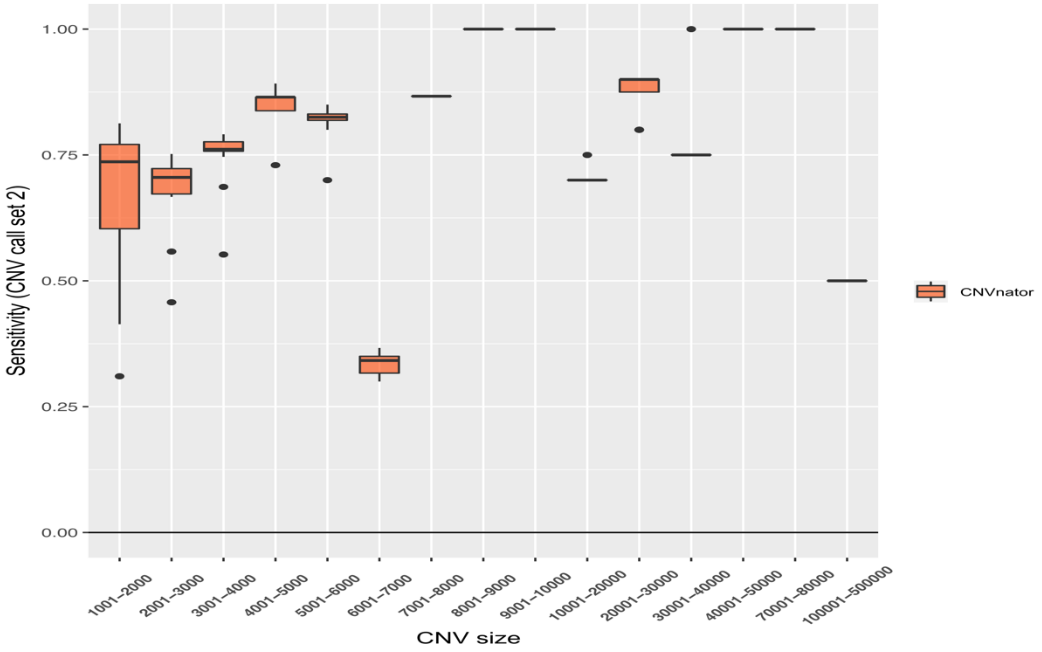

Supplementary Figure 5 Sensitivity of CNV detection in down-sampling samples of NA12878-1 by BreakDancer (CNV call set 2)

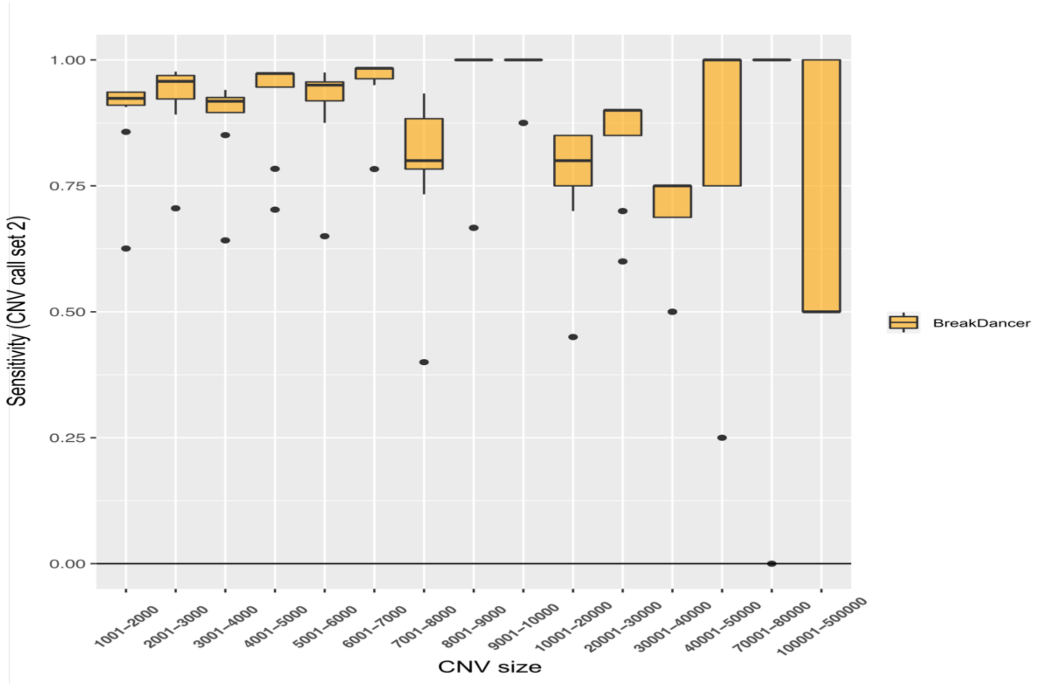

Supplementary Figure 6 Sensitivity of CNV detection in down-sampling samples of NA12878-1 by LUMPY (CNV call set 2)

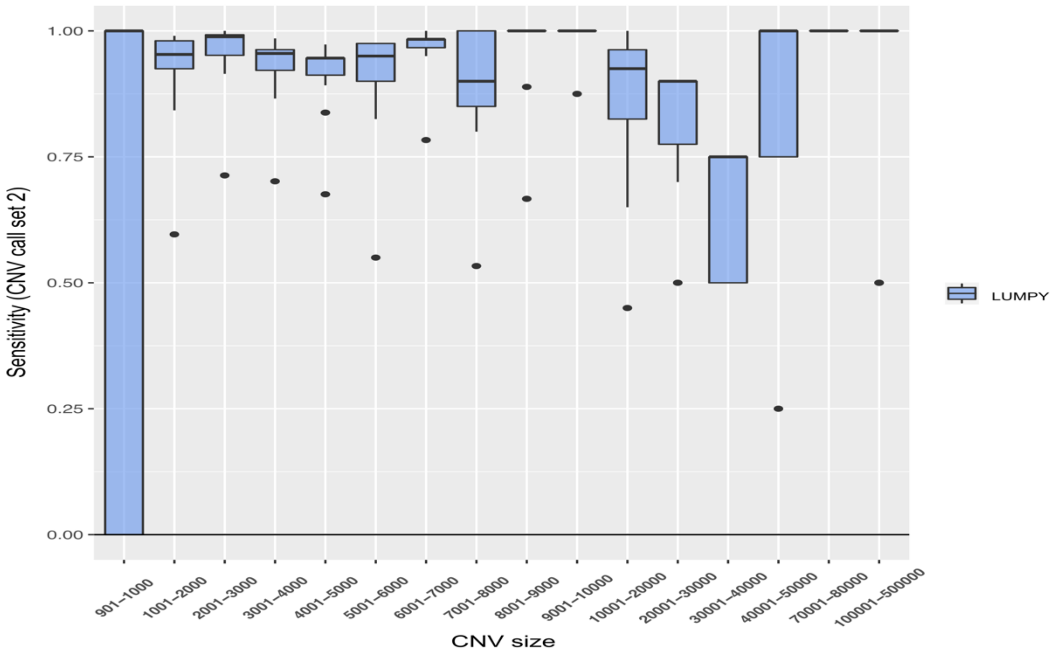

Supplementary Figure 7 Sensitivity of CNV detection in down-sampling samples of NA12878-1 by CNVnator (CNV call set 3)

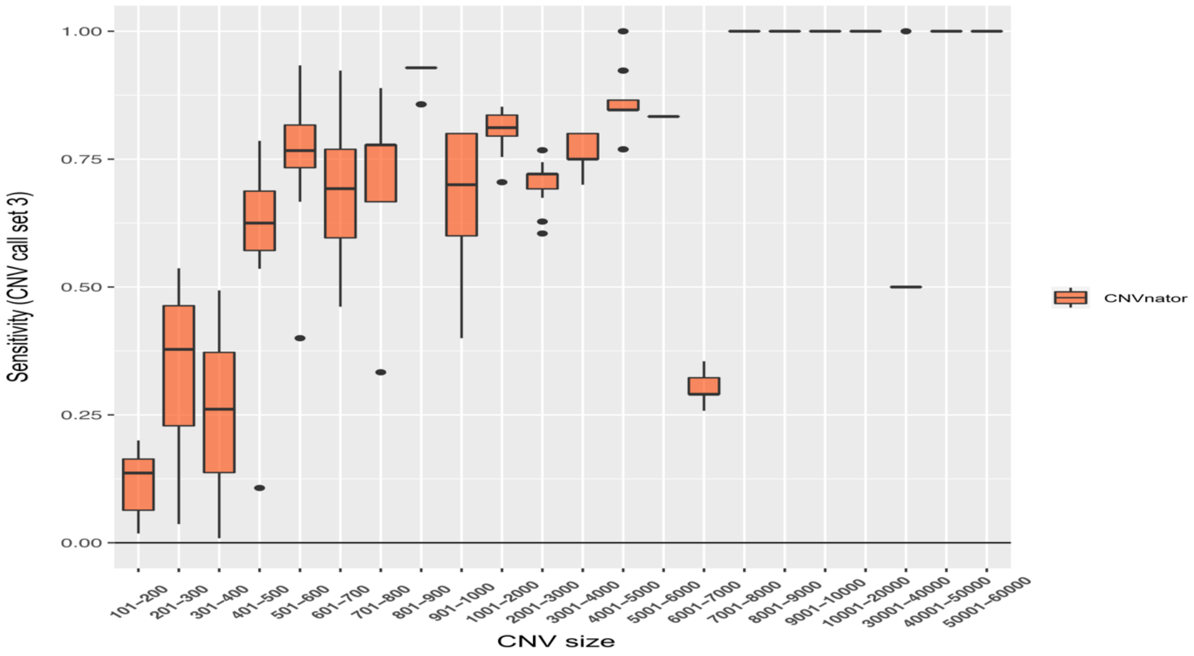

Supplementary Figure 8 Sensitivity of CNV detection in down-sampling samples of NA12878-1 by BreakDancer (CNV call set 3)

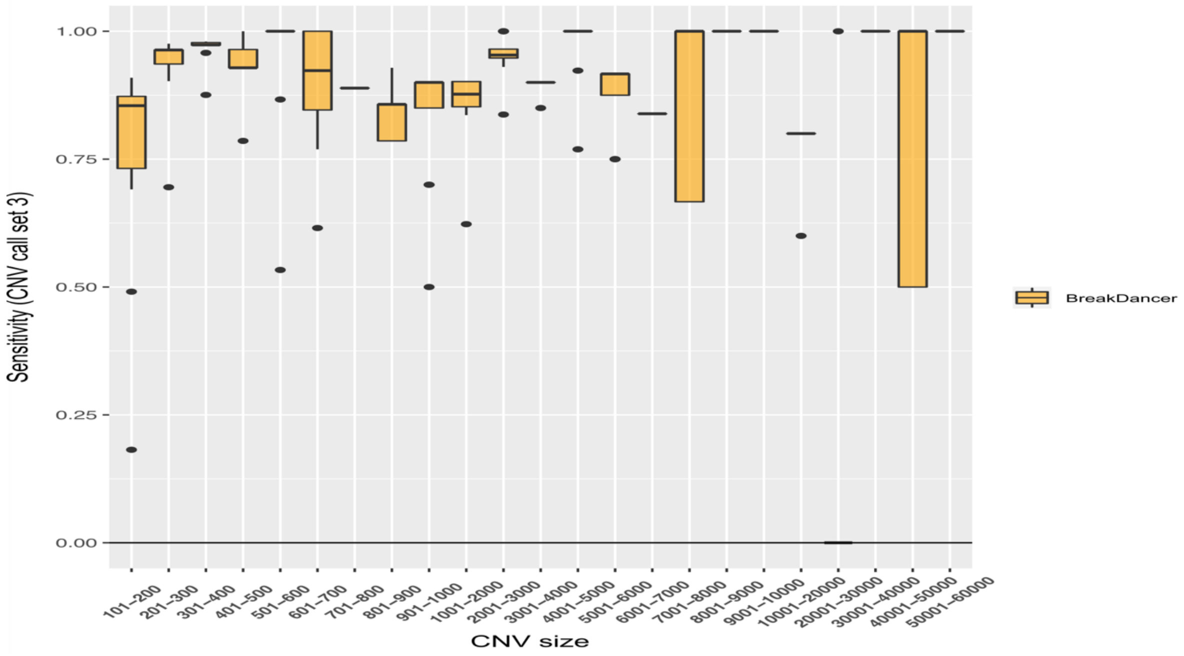

Supplementary Figure 9 Sensitivity of CNV detection in down-sampling samples of NA12878-1 by LUMPY (CNV call set 3)

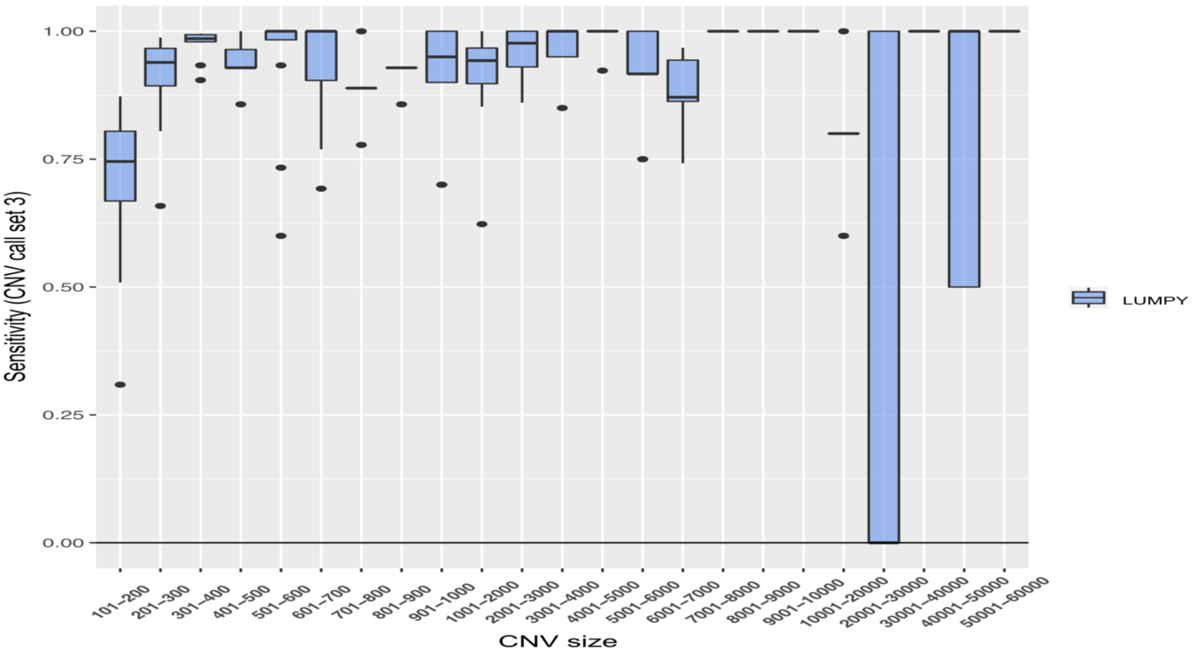

Supplementary Figure 10 Sensitivity of deletion detection in down-sampling samples of NA12878-1 by CNVnator (CNV call set 1)

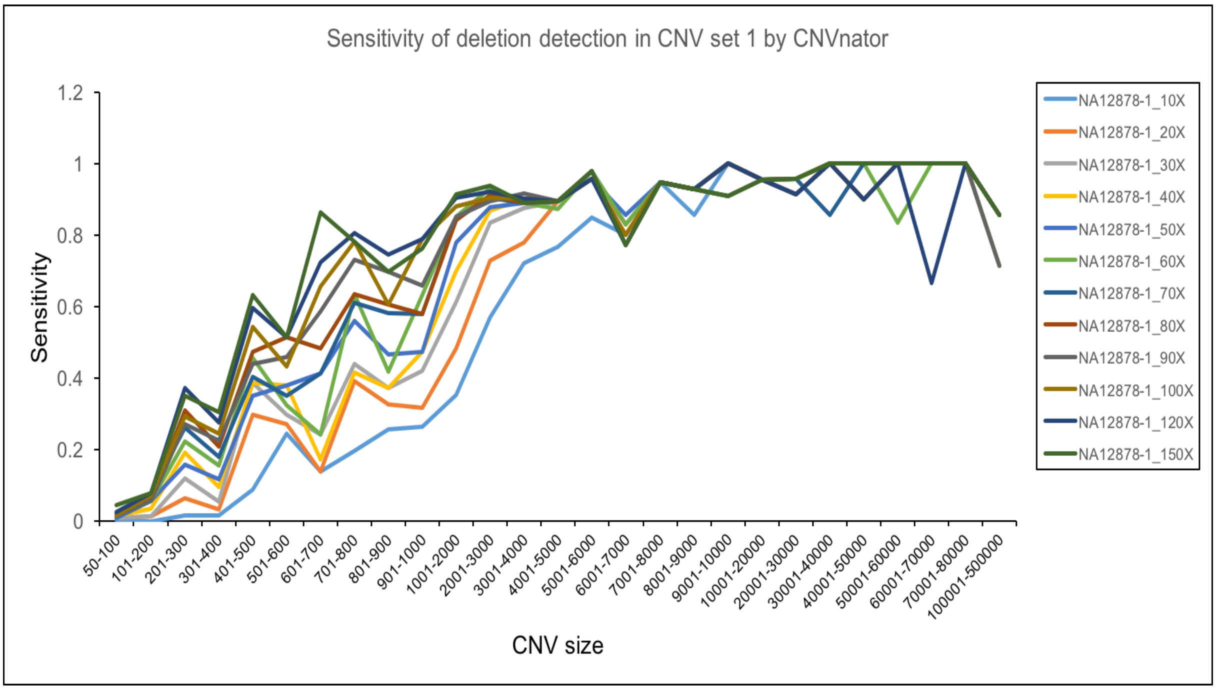

Supplementary Figure 11 DP and GQ distribution for NA12878-2_120X (WES) and NA12878-1_40X (WGS) in the regions of the human genome covered by WES

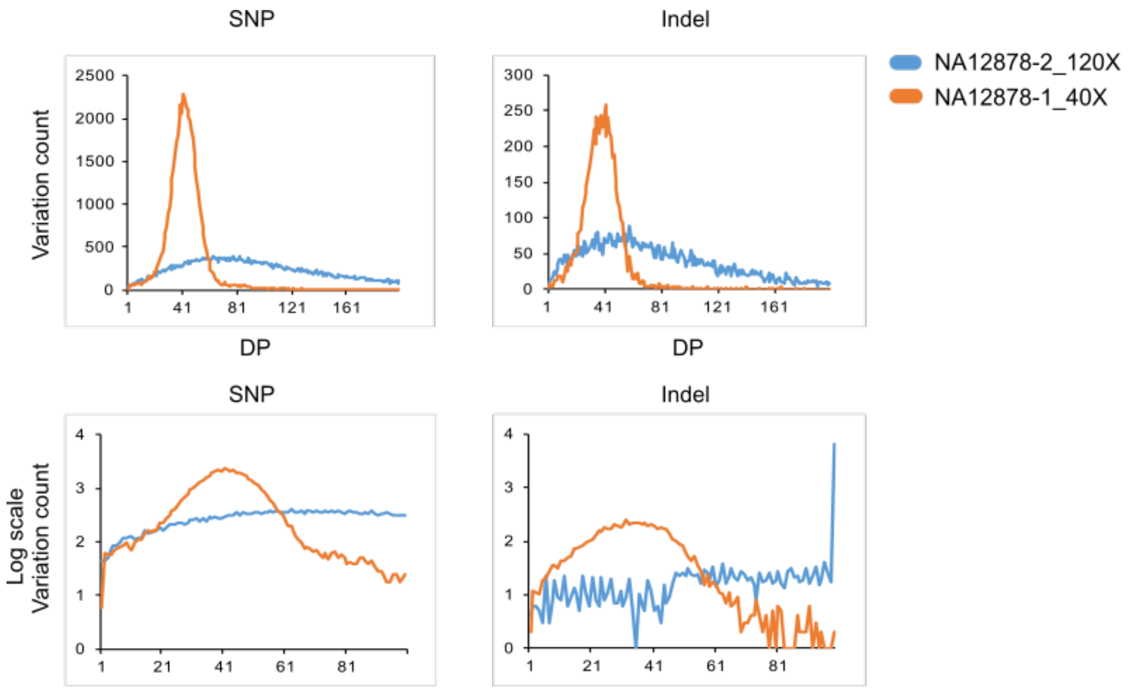

Supplement: Supplementary file 1 — Additional file 1. Supplementary Tables. [file 12920_2021_948_MOESM1_ESM.pdf]
